# Does education moderate gender disparities in later-life memory function? A cross-national comparison of harmonized cognitive assessment protocols in the United States and India

**DOI:** 10.1002/alz.13404

**Published:** 2023-07-25

**Authors:** Ashly C. Westrick, Justina Avila-Rieger, Alden L. Gross, Timothy Hohman, Jet M. J. Vonk, Laura B. Zahodne, Lindsay C. Kobayashi

**Affiliations:** 1 Department of Epidemiology, Center for Social Epidemiology and Population Health, University of Michigan: School of Public Health, Ann Arbor, Michigan, USA; 2 Gertrude H. Sergievsky Center and the Taub Institute for Research in Aging and Alzheimer’s Disease, Columbia University, New York, New York, USA; 3 Department of Epidemiology, Johns Hopkins Bloomberg School of Public Health, Center on Aging and Health, Johns Hopkins University, Baltimore, Maryland, USA; 4 Vanderbilt Memory & Alzheimer’s Center, Vanderbilt University Medical Center, Nashville, Tennessee, USA; 5 Vanderbilt Genetics Institute, Vanderbilt University Medical Center, Nashville, Tennessee, USA; 6 Department of Neurology, Memory and Aging Center, University of California San Francisco (UCSF), San Francisco, California, USA; 7 Department of Psychology, University of Michigan, Ann Arbor, Michigan, USA

**Keywords:** cross-national comparison, education, gender, memory

## Abstract

**INTRODUCTION:**

We compared gender disparities in later-life memory, overall and by education, in India and the United States (US).

**METHODS:**

Data (*N* = 7443) were from harmonized cognitive assessment protocols (HCAPs) in the Longitudinal Aging Study of India-Diagnostic Assessment of Dementia (LASI-DAD; *N* = 4096; 2017–19) and US Health and Retirement Study HCAP (HRS-HCAP; *N* = 3347; 2016–17). We derived harmonized memory factors from each study using confirmatory factor analysis. We used multivariable-adjusted linear regression to compare gender disparities in memory function between countries, overall and by education.

**RESULTS:**

In the United States, older women had better memory than older men (0.28 SD-unit difference; 95% CI: 0.22, 0.35). In India, older women had worse memory than older men (−0.15 SD-unit difference; 95% CI: −0.20, −0.10), which attenuated with increasing education and literacy.

**CONCLUSION:**

We observed gender disparities in memory in India that were not present in the United States, and which dissipated with education and literacy.

## 1 | INTRODUCTION

The United States (US) and India are both experiencing rapidly aging populations.^1,2^ By 2050, 20% of the United States and 19% of the Indian population will be over aged 65 and 60, respectively.^1,2^ The prevalence and population burden of cognitive impairment and dementia are thus expected to rapidly increase in both countries in the coming decades. Cross-national comparisons present a unique opportunity to investigate reasons for differences in patterns of cognitive outcomes across countries, which can serve to inform strategies to reduce the global burden of dementia. The US and India vary substantially in terms of economic, health, and cultural factors that are purported risk factors for dementia, including early-life education, literacy, employment, and environment risks.^3^ Despite the need to understand cognitive aging globally, there’s limited comparative research on cognitive aging outcomes in the United States and India.^4,5^

Prior cross-national research on cognitive aging has shown that women living in low- and middle-income countries have a cognitive disadvantage compared to their male counterparts, while this disparity is less pronounced in high-income countries.^5–7^ Studies in the US have consistently found that women have on average stronger verbal memory skills than men, but men have on average stronger visuospatial skills than women.^8,9^ Another US study showed that, despite women having higher baseline cognitive scores than men, they exhibit faster rates of decline in global cognition and executive function than men as they aged.^10^ Studies in India have found that older women tend to have worse cognitive outcomes compared to men across all cognitive domains,^7,11,12^ with a wider gap amongst the oldest age groups.^11^ Further, at equivalent levels of education, Indian women tend to perform as well as or better than their male counterparts on cognitive tests that did not require literacy, while women tended to perform worse than men on cognitive tests that required literacy.^11^ These gender differences in cognitive outcomes across the US and India may thus relate to differences in societal gender inequities across countries.^13^ According to the United Nations, in 2021, the Gender Inequality Index (range 0–1), a composite measure of gender inequality with higher values indicating greater inequality among men and women, was 0.49 for India while it was 0.18 for the United States.^14^

While gender differences in cognitive aging are complex and could be influenced by biological or genetic differences associated with sex,^9,15,16^ a growing body of research suggests that they are largely influenced by societal gender inequities. In particular, gender-based disparities in access to education could impact later-life gender disparities in cognition, as education is a strong protective factor against dementia risk.^17,18^ According to the 2011 Indian Census, there was a gap in upper secondary school educational attainment between men and women across all ages, whereby 70% of women had no upper secondary education compared to 58% of men.^19–21^ To-date, there is limited research on how the cognitive function of older Indian adults may differ from that of older American adults by gender and educational attainment. This evidence gap limits our understanding of gender-based disparities in cognitive aging outcomes within and across countries.

We aimed to compare gender differences in later-life memory, overall and by education, using harmonized data from two nationally-representative studies of aging: the US Health and Retirement Study Harmonized Cognitive Assessment Protocol (HRS-HCAP) and the Longitudinal Aging Study of India-Diagnostic Assessment of Dementia (LASI-DAD) in India. This study builds on prior evidence that education may mediate the relationship between gender and later-life cognition,^22–24^ as few studies have directly examined the magnitudes of gender differences in later-life cognition across levels of education, and across countries with differing levels of societal gender inequities and economic development. We hypothesized that we would observe a gender disparity in later-life memory function in India but not in the United States, whereby women experience a memory disadvantage relative to men, but that this disparity would potentially be ameliorated at higher levels of educational attainment.

## 2 | METHODS

### 2.1 | Data sources

Data were from the HRS-HCAP in the United States and the LASI-DAD in India.^25–28^

The HRS is an ongoing nationally representative longitudinal study on the health, economic, and social well-being of over 20,000 adults aged >50 in the United States that began in 1992.^26^ The HRS-HCAP aims to comprehensively measure cognitive function using a neuropsychological protocol.^28^ A total of 3496 HRS participants aged ≥65 years who completed the 2016 core interview and venous blood collection were randomly selected and participated in the HRS-HCAP. For this study of episodic memory, 149 (4.1%) participants with only proxy interviews were excluded. Participants were evaluated in their preferred language (English or Spanish).

The LASI is an ongoing nationally representative longitudinal study on the health, economic, and social well-being of over 70,000 adults aged >45 in India that began in 2017.^25^ The design and measures of the LASI are harmonized with those of the HRS, as it is an International Partner Study of the HRS. The LASI-DAD uses the same suite of cognitive function measures as the HRS-HCAP, with necessary language translations and adaptations for literacy and cultural appropriateness.^27^ A total of 4096 LASI participants aged ≥60 years from 14 Indian States and Union Territories were selected and participated in LASI-DAD, which was fielded in three phases from 2017 to 2019. The LASI-DAD oversampled individuals at high risk of cognitive impairment and sampling weights were created to account for these differential selection probabilities.^27^ Participants were evaluated in their local language (Hindi, Kannada, Malayalam, Gujarati, Tamil, Punjabi, Urdu, Bengali, Assamese, Odiya, Marathi, or Telugu).

### 2.2 | Measures

#### 2.2.1 | Gender

In the HRS-HCAP, gender is self-reported by the respondents. In the LASI-DAD, the interviewer indicates the respondent’s gender.

#### 2.2.2 | Memory

Using an item banking approach within an item response theory (IRT) framework, we derived a factor score representing memory function that was harmonized to be on the same scale across the HRS-HCAP and LASI-DAD samples. Using a cultural neuropsychological approach to pre-statistical harmonization, we identified memory test items that were common and unique due to adaptations across the two studies.^29^ We deemed all memory test items to be comparable between the studies, except for the Consortium to Establish a Registry for Alzheimer’s Disease (CERAD) word learning tasks. Differential item functioning (DIF) was assessed in previous analysis and deemed negligible. In confirmatory factor analysis models, memory test items deemed common across the HRS-HCAP and LASI-DAD were constrained to have the same model parameters across the two studies, while unique items had freely estimated parameters. Model fit was assessed using a combination of the Root Mean Square Error of Approximation (RMSEA), Comparative Fit Index (CFI), and Standardized Root Mean Residual (SRMR) (Table S1). To facilitate comparability, each study’s memory function factor score was scaled to the HRS-HCAP distribution, which had a mean of 0 and a standard deviation of 1. The pre-statistical^29,30^ and statistical procedures^29,31^ are described in further detail elsewhere.

#### 2.2.3 | Education

We harmonized educational attainment according to the 2011 International Standard Classification of Education (ISCED), which provides an internationally standardized classification of educational attainment.^32^ Educational attainment was measured in the HRS using the years of education and respondent’s highest degree (no degree, GED, high school/GED, Associates degree, Bachelor’s degree, master’s degree, professional degree) and in the LASI as less than primary school, primary school, middle school, secondary school, higher secondary school, diploma/certificate holders, graduate degree (BA, BSc), post-graduate degree, professional course/degree. We cross-walked these raw variables with the 2011 ISCED to generate a harmonized educational attainment variable with categories of: none or early education, primary education, lower secondary education, upper secondary education, and any college. We modified the original ISCED 2011 classifications to include an additional category of “no formal education,” a category which was largely applicable to the LASI-DAD sample.

#### 2.2.4 | Covariates

We selected as covariates potential confounders of the relationships between each gender and education predictor and later-life memory function. Such covariates would have to arise early in life, prior to the completion of education. These were: age (continuous), age-squared to account for non-linear age differences in memory (continuous), minority group status (minority vs. non-minority), mother’s education (no formal education vs. primary education or higher), and father’s education (no formal education vs. primary education or higher). Minority group status was a dichotomous variable in the HRS-HCAP and LASI-DAD studies. Due to differences in the social constructs of race and ethnicity across the two countries and the need to create harmonized variables for pooled analyses, we established a minority group classification based on historically marginalized racial or ethnic groups within each of the United States and India. For the HRS-HCAP, we categorized minority group status based on self-identified race/ethnicity and those who identified as non-Hispanic White were classified as “non-minority” and those who did not identify as non-Hispanic White were classified as “minority.” To create a comparable and appropriate variable to represent minority group in India, we used the caste variable, which has been used in previous population-based studies in India.^11,12,33,34^ The Indian caste system is a religion-based hierarchical social structure which divides Indian society into groups based on occupations.^35^ Caste in LASI-DAD was categorized as Scheduled Tribe (ST), Scheduled Caste (SC), Other Backward Class (OBC), and others. In LASI-DAD, those who identified as SC or ST were classified as “minority,” as these groups occupy the lowest rank in the caste hierarchy.^36^ Those who identified as OBC or other caste categories were classified as “non-minority” as they have higher status in the caste hierarchy.

### 2.3 | Statistical analyses

We described characteristics of the HRS-HCAP and LASI-DAD samples by gender. Because very few participants in the HRS-HCAP had no formal education, we pooled the no education/early education category with the primary education category and used this combined category as the reference group in modeling. First, to examine whether gender differences in memory function varied by country, we estimated country-specific linear regression models with memory regressed on gender, while adjusting for age and age-squared. Second, to examine whether these gender differences were modified by educational attainment, we added education and a gender*education interaction term to these models. HRS-HCAP and LASI-DAD sampling weights were applied to all models. To examine the potential impact of literacy differences within the LASI-DAD study sample on our results, we conducted a sensitivity analysis where we re-estimated our models within the literate sub-sample of LASI-DAD (*N* = 1777; 43.4%) and all HRS-HCAP participants. All analyses were conducted using Stata version 16.1 and R statistical software (version 4.0.5).

## 3 | RESULTS

### 3.1 | Sample characteristics

Our sample consisted of 7443 participants: 4096 in the LASI-DAD and 3347 in the HRS-HCAP. There were more women than men in both cohorts (LASI-DAD: 53.9% and HRS-HCAP: 60.3%; Table 1). The mean age of participants in the HRS-HCAP was 76 years compared to 68 years in the LASI-DAD (Table 1). While few HRS-HCAP participants had no formal or early education (0.4% of men and 0.8% of women), nearly half of men (47%) and three-quarters of women (76%) in India had no formal or early education (Table 1). Most HRS-HCAP participants had parents with at least a primary education, while few LASI-DAD participants reported having parents with at least a primary education (11% of men and 9% of women had a mother with at least a primary education; 22% of both men and women had a father with at least a primary education).

### 3.2 | Regression analyses

**FIGURE 1:**
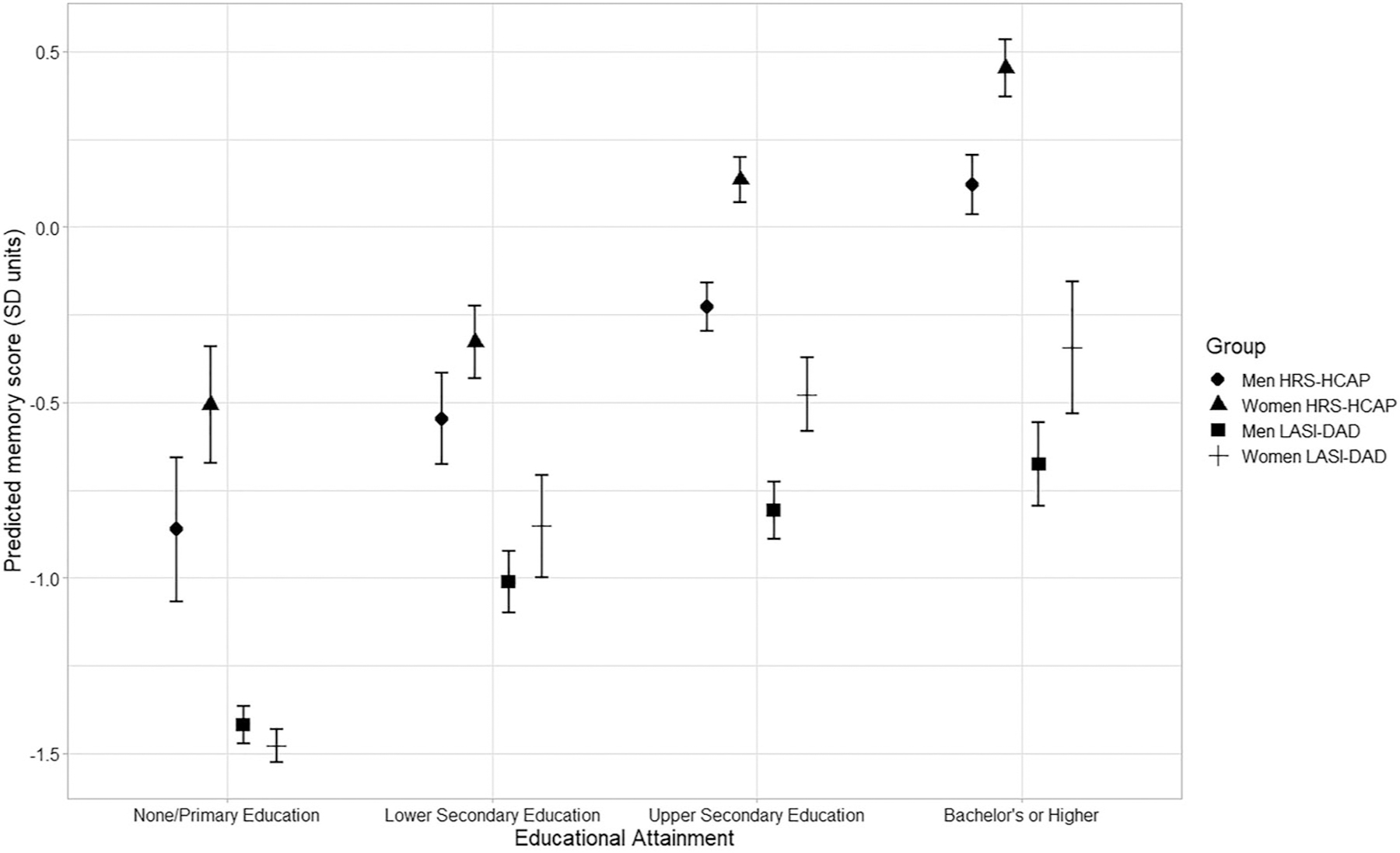
Predicted memory scores in SD units for men and women in the HRS-HCAP (*N* = 3347) and LASI-DAD (*N* = 4096), by educational attainment.

Controlling for age and age-squared, older women had worse memory than older men in India, on average (−0.15 SD units; 95% CI: −0.20, −0.10), while older women had better memory than older men in the United States (0.28 SD units; 95% CI: 0.22, 0.35; Table 2). Subsequent models with the gender*education interaction identified that, among those with at least lower secondary education, women in India experienced a later-life memory advantage over men, an advantage that increased with greater levels of educational attainment (Table 2; Wald *p* < 0.001). In both the United States and India, women with upper secondary education or a Bachelor’s degree or higher had better memory scores than men with similar levels of education (Figure 1). Cross-nationally, men and women in the HRS-HCAP experienced better memory within each educational attainment group compared to men and women in the LASI-DAD (Figure 1).

### 3.3 | Sensitivity analyses: HRS-HCAP versus LASI-DAD literates

**FIGURE 2:**
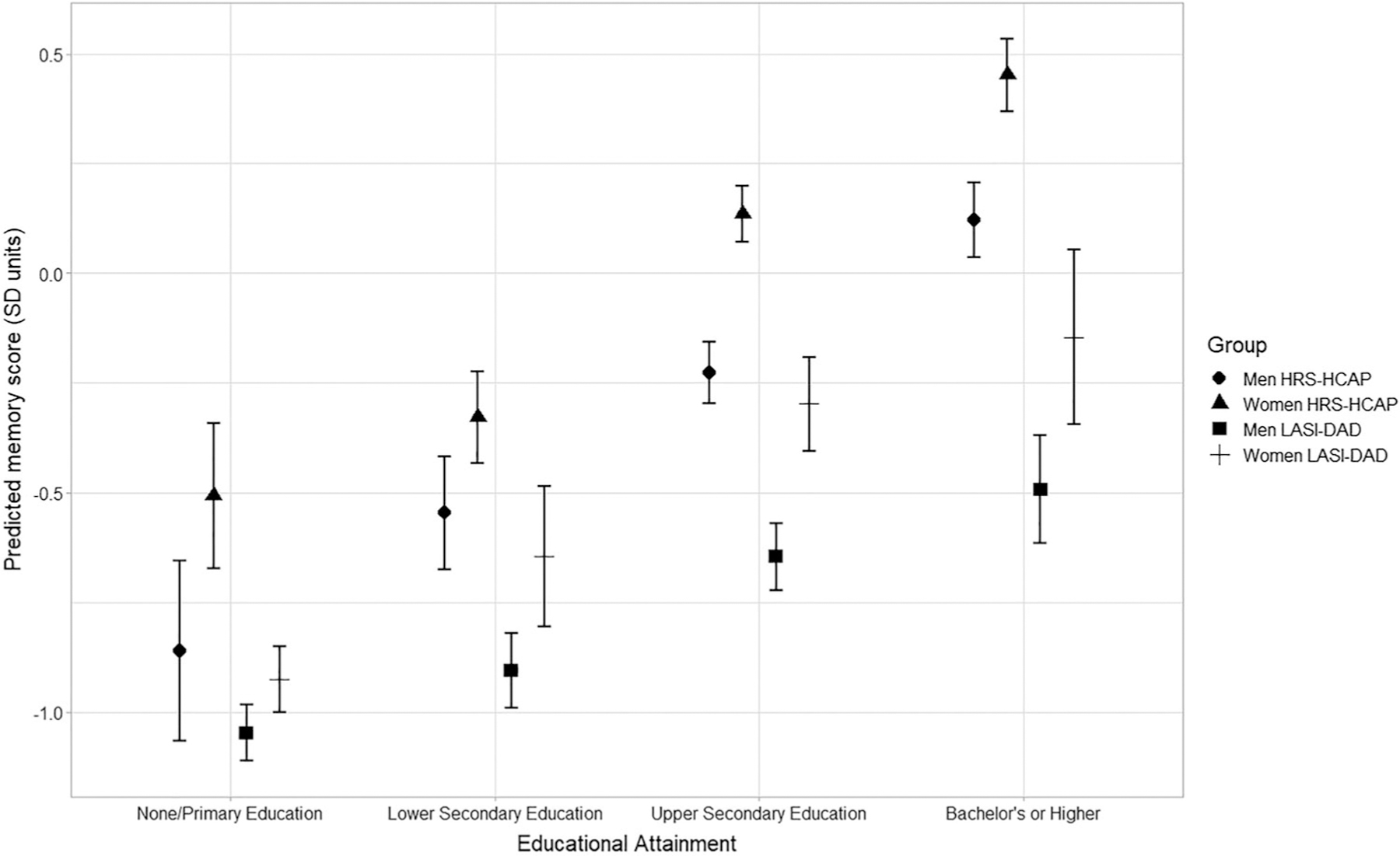
Predicted memory scores in SD units for literate men and women in the HRS-HCAP (*N* = 3347) and LASI-DAD (*N* = 1777), by educational attainment.

We conducted a sensitivity analysis restricting the LASI-DAD sample to only those who were literate (*n* = 1777; 43.4%). After adjusting for age and age-squared, literate women in the LASI-DAD had higher memory scores, on average, than literate men (0.16 SD units; 95% CI: 0.09, 0.23; *p* < 0.001; Table 3). In this analysis among the literate LASI-DAD sub-sample, higher educational attainment was associated with better memory among men and women in both countries (Table 3; Figure 2).

Compared to our main results, the cross-national difference in memory scores within each educational attainment category was attenuated.

## 4 | DISCUSSION

In this large, population-based and harmonized study of older adults in the United States and India, we observed gender disparities in later-life memory function in India with older women in India having a disadvantage compared to older men that were not present in the United States, and which dissipated with increasing educational attainment and literacy. Given that biological sex differences that may affect memory are not considered to vary across these populations, these cross-national variations in the magnitude and direction of gender disparities in later-life memory may be due to differences in societal gender inequities between the United States and India that differentially shape later-life cognitive health among men and women.

### 4.1 | Comparison to existing literature

Few studies have explored cross-national differences in gender disparities in later-life memory function. One recent study investigated sex differences in the United States compared to Mexico, Brazil, China, and India.^23^ The authors found that females had better memory performance in the United States, Mexico, and Brazil but worse in China and India. Similar to our study, the authors found that, when adjusting for education, there were no sex differences in memory function among older adults in India.^23^ Our study builds on these results by using a cultural neuropsychological approach to harmonized measures of memory scores across the United States and India. The use of cross-national comparisons allow for an in depth analysis of disparities in later-life cognitive function within different cultural, economic, and societal contexts.

Our results are consistent with previous evidence from low- and middle-income settings that have reported that older women perform on average worse on memory function measures than older men,^37–39^ in contrast to high-income settings, where older women typically outperform older men.^40,41^ Consistent with our findings, previous research in India has found substantial gender gaps in later-life cognition, with older Indian women having worse memory function than older Indian men.^11,33^ The current study builds on previous research by demonstrating that, across two countries with different distributions of educational attainment and differing levels of societal gender inequity, there is either no gender disparity or a later-life memory advantage among women relative to men with higher education. Education is crucial for cognitive development in early-life and has been identified as a source of cognitive reserve and a strong protective factor for later-life cognitive outcomes.^42–44^ However, societal expectations of gender roles have historically manifested in reduced access to education for women.^45^ Bonsang and colleagues found that, across 27 countries, women had better cognitive function than men in countries characterized by more equal gender-role attitudes.^13^ Furthermore, they found that greater educational attainment and labor-force participation among women partially mediated the relationship between gender-role attitudes and later-life cognitive function. Countries such as India with stronger gender role attitudes, particularly among older generations, have restricted educational and labor force opportunities for women, which are sources of cognitive stimulation that can improve and strengthen cognitive function.^22,46,47^ Indeed, a study by Jain et al., found that, in Indian regions with higher levels of gender inequality and discrimination against women, older women’s cognitive disadvantage relative to older men was worse.^33^

Our findings suggest that improving women’s access to education early in life may help reduce gender disparities in later-life memory function. Multiple studies have found the importance of investing in women’s education on fertility, child mortality, empowerment, financial improvements, and later-life health including cognitive aging.^22,48,49^ However, an alternative explanation is that older women in India who achieved higher educational attainment may have had experiences and social privileges in earlier life that enabled them to achieve higher educational attainment, and which also may have protected their cognitive health in later-life. Because the barriers to educational system entry and achievement have historically been much higher for women relative to men in India,^50^ women in the LASI-DAD sample who achieved higher education may have had greater early-life social advantage than their men counterparts. We adjusted for early-life factors as best as possible in this study, but we were limited to parental education and minority group status as markers of early-life socioeconomic status. There may be residual confounding in our estimates by other early-life social and health-related factors, which should be investigated in future studies. Future studies should also elucidate the role of early-life gender inequities in shaping later-life cognitive outcomes, particularly across different social and economic settings. It will also be important to repeat this analysis in later-born cohorts due to the shifting landscape of education and social inequalities in India and the United States.

Other limitations include that we could not assess the longitudinal rate of cognitive decline due to the cross-sectional nature of the HCAPs implemented to-date in the HRS and LASI. When longitudinal data are available, future studies should evaluate differences in changes in cognitive function over time. Additionally, we were limited in exploring results across more nuanced racial, ethnic, and caste-based minority population subgroups. Previous research has shown significant disparities in memory aging experienced by minority populations living across different global regions.^51^ Furthermore, we were not able to assess differences across regions of India with differing degrees of educational opportunities for women. Analyses of sub-national variations in gender disparities in memory aging are an important area of future study. Finally, selective survival bias may impact our results, as older adults in both countries had to survive to the time of study enrollment to take part in the LASI-DAD and HRS-HCAP. Because these countries have different life expectancies, the potential magnitudes of selective survival bias in the LASI-DAD and HRS-HCAP may differ, despite the lower minimum age of eligibility for LASI-DAD than HRS-HCAP. Future research should determine how selective survival could affect these results, as well as results of other cross-national comparisons of aging populations.

Our study has several notable strengths. We used data from two large population-based and harmonized studies of older adults in the United States and India. Memory function was assessed comprehensively using high-quality neuropsychological assessments from the HCAP, an innovative set of adapted instruments that is designed specifically for the purpose of cross-national comparisons of cognitive function. We used a cultural neuropsychological approach to identify common and unique items across HCAP administrations in each country, and we used statistically harmonized measures of memory function that were of good fit to the data in both countries.

## 5 | CONCLUSION

In this cross-national comparison of gender disparities in later-life memory function in the United States and India, we observed strong gender disparities in later-life memory function in India that were not present in the United States, and which dissipated with increasing educational attainment and literacy. While early-life selection into education cannot be ruled out as an explanation, our findings suggest that increasing educational access for women in India may help to reduce later-life gender disparities in cognitive aging outcomes in this populous and rapidly aging country.

## Supplementary Material

Supplementary Material

## Figures and Tables

**TABLE 1:** Characteristics of the samples, LASI-DAD (*n* = 4096) and HRS-HCAP (*n* = 3347)

	LASI-DAD		HRS-HCAP	
Characteristic	Women (*n* = 2207)	Men (*n* = 1889)	Women (*n* = 2020)	Men (*n* = 1327)
Age, *years* (mean, SD, range)	69.3 (7.8, 60–105)	70.2 (7.4, 60–103)	76.7 (7.6, 64–102)	76.4 (7.3, 64–101)
Education, *years* (mean, SD, range)	0.53 (1.2, 0–7)	1.42 (1.8, 0–7)	3.55 (1.8, 0–7)	3.86 (1.8, 0–7)
No formal education or early childhood education (N; %)	1,674; 76%	884; 47%	17; 0.8%	5; 0.4%
Primary education	226; 10%	301; 16%	78; 4%	53; 4%
Lower secondary	100; 5%	214; 11%	297; 15%	157; 12%
Upper secondary	165; 7%	340; 18%	1,101; 54%	672; 51%
Any college	42; 2%	150; 8%	526; 26%	439; 33%
Minority group status (N; % minority)	507; 23%	449; 24%	626; 31%	337; 25%
Mother’s education (N; % with at least primary)	169; 9%	239; 11%	1949; 96%	1288; 97%
Father’s education (N; % with at least primary)	496; 22%	417; 22%	1945; 96%	1289; 97%

Abbreviations: HRS-HCAP, Health and Retirement Study Harmonized Cognitive Assessment Protocol; LASI-DAD, Longitudinal Aging Study of India-Diagnostic Assessment of Dementia.

**TABLE 2:** Associations between gender, educational attainment, and memory function, LASI-DAD (*n* = 4096) and HRS-HCAP (*n* = 3347)

	LASI-DAD		HRS-HCAP	
Parameter	Coef.	95% CI	Coef.	95% CI
Model 1*				
Gender (women vs. men)	−0.15	(−0.20, −0.10)	0.28	(0.22, 0.35)
Model 2**				
Gender (women vs. men)	−0.06	(−0.11, −0.01)	0.36	(0.10, 0.63)
*Education*				
None/early or primary (ref.)	–	–	–	–
Lower secondary	0.42	(0.33, 0.51)	0.29	(0.05, 0.54)
Upper secondary	0.63	(0.54, 0.72)	0.57	(0.34, 0.80)
Bachelor’s or higher	0.76	(0.63, 0.89)	0.91	(0.68, 1.14)
Gender*education				
Gender × lower secondary	0.23	(0.06, 0.40)	−0.15	(−0.45, 0.16)
Gender × upper secondary	0.40	(0.27, 0.54)	0.01	(−0.26, 0.28)
Gender × Bachelor’s or higher	0.40	(0.17, 0.63)	−0.03	(−0.31, 0.26)

Abbreviations: HRS-HCAP, Health and Retirement Study Harmonized Cognitive Assessment Protocol; LASI-DAD, Longitudinal Aging Study of India-Diagnostic Assessment of Dementia.

* Adjusted for age, age-squared, with sampling weights.

** Adjusted for age, age-squared, mother’s and father’s education, and minority status, with sampling weights.

**TABLE 3:** Sensitivity analysis of the association between gender, educational attainment, and memory function, restricting to literate participants in LASI-DAD (*n* = 1777) and HRS-HCAP (*n* = 3347)

	LASI-DAD		HRS-HCAP	
	Coef	95% CI	Coef	95% CI
Model 1*				
Gender (women vs. men)	0.16	(0.09, 0.23)	0.28	(0.22, 0.35)
Model 2**				
Gender (women vs. men)	0.12	(0.02, 0.22)	0.36	(0.10, 0.63)
*Education*				
None/early or Primary (ref.)	–	–	–	–
Lower secondary	0.14	(0.04, 0.25)	0.29	(0.05, 0.54)
Upper secondary-short tertiary	0.40	(0.30, 0.50)	0.57	(0.34, 0.79)
Bachelor’s or higher	0.55	(0.41, 0.70)	0.91	(0.68, 1.14)
Gender*education				
Gender × lower secondary	0.14	(−0.06, 0.34)	−0.15	(−0.45, 0.16)
Gender × Upper secondary-short tertiary	0.23	(0.07, 0.39)	0.01	(−0.26, 0.28)
Gender × Bachelor’s or higher	0.22	(−0.02, 0.47)	−0.03	(−0.31, 0.26)

Abbreviations: HRS-HCAP, Health and Retirement Study Harmonized Cognitive Assessment Protocol; LASI-DAD, Longitudinal Aging Study of India-Diagnostic Assessment of Dementia.

* Adjusted for age, age-squared, with sampling weights.

** Adjusted for age, age-squared, mother’s and father’s education, and ethnic minority status, with sampling weights.

## RESEARCH IN CONTEXT

### 1. Systematic review:

The authors reviewed the literature using traditional (e.g., PubMed) sources. Because there is limited comparative research on the cognitive aging outcomes of older adults in the United States and India, research describing gender differences in later-life cognitive outcomes in the United States and India as well as literature on the influence of education on later-life cognitive outcomes was used to inform hypotheses in the current study.

### 2. Interpretation:

We observed strong gender disparities in later-life memory function in India that were not present in the United States, and which dissipated with increasing educational attainment and literacy.

### 3. Future directions:

This study takes an important step in understanding the potential modifying role of education in gender disparities in later-life memory function across the United States and India. Additional studies are warranted to expand evidence on the role of early-life gender inequities in shaping later-life cognitive outcomes, particularly across different social and economic settings.
