# Supplementary material for: Does education moderate gender disparities in later-life memory function? A cross-national comparison of harmonized cognitive assessment protocols in the United States and India
Source: Alzheimers Dement. Author manuscript; Available in PMC 2024 Feb 16. (PMC10808282; doi:10.1002/alz.13404)
Supplement: Supplementary Material [file NIHMS1930769-supplement-Supplementary_Material.docx]

| **Supplemental Table 1.** Fit statistics for memory in the LASI-DAD (n = 4,096) and HRS-HCAP (n = 3,347) | | | | |
| --- | --- | --- | --- | --- |
| **Cognition Domain** | **Study** | **RMSEA** | **CFI** | **SRMR** |
| Memory | HRS | 0.045 | 0.980 | 0.023 |
| Memory | LASI-DAD – illiterate | 0.049 | 0.965 | 0.031 |
| Memory | LASI-DAD – literate | 0.046 | 0.978 | 0.027 |
